# Radiological anatomy of the trochlear spine and associated bony structures around the superior oblique tendon: a CT-based study

**DOI:** 10.1007/s12565-025-00871-0

**Published:** 2025-07-15

**Authors:** Denise Bonente, Tiziana Tamborrino, Niccolò Fagni, Sandra Bracco, Sara Leonini, Sara Ottolenghi, Virginia Barone, Eugenio Bertelli

**Affiliations:** 1 Department of Molecular and Developmental Medicine, University of Siena, Via Aldo Moro, 53100 Siena, Italy; 2 Department of Life Sciences, University of Siena, Via A. Moro, 2-53100 Siena, Italy; 3 Otorinolaringology, AOUS (Azienda Ospedaliero-Universitaria Senese), Siena, Italy; 4 Unit of Interventional Neuroradiology, Azienda Ospedaliero Universitaria Senese (AOUS), University of Siena, Policlinico “Santa Maria Alle Scotte”, Siena, Italy; 5 Radiology Department, Santa Corona Hospital, Pietra Ligure, Italy

**Keywords:** Orbit, Superior oblique muscle, Eye, Computed tomography, Trochlea

## Abstract

The superior oblique muscle tendon is known to bend in the anterior orbit around a cartilaginous trochlea. The site where the tendon bends is frequently interested by the presence of a small depression, the trochlear fovea, and/or the trochlear spine. Exact topography, size and frequency of these items are still undetermined. For this purpose, we studied 120 orbits of individuals that underwent computed tomography for pathologies not involving the anterior orbit. We detected the presence of the trochlear spine in 10% of orbits and we determined its location and size. We also observed the presence of two tubercles (TT_1_ and TT_2_), with distinct positions relative to the tendon. TT_1_ was present in 5% of orbits and was located on the same spot of the spine differing from the latter only for its morphology. TT_2_ lied in a more advanced position and it was rarer (1,67% of orbits). The spine and the first type of tubercle were located above and behind the tendon reflection; the second tubercle lied below and ahead of the tendon reflection. A distinct trochlear fovea was detected in 25.83% of orbits and lied 3.42 ± 0.97 mm behind the orbital rim. Fovea diameters were 4.16 ± 1.08 mm × 3.84 ± 0.97 mm. In conclusion we demonstrate that in the anterior orbit a bony process is present in at least 15% or orbits. It is a note of interest for strabismus surgery when it is necessary to intervene on the superior oblique muscle or when it is needed access to the medial orbital wall.

## Introduction

The superior oblique muscle is one of the six extraocular muscles that supply the force to generate eye movements. Along with the inferior oblique muscle, its tendon approaches the eyeball moving laterally and backward. However, in contrast to the inferior one which arises from the anterior orbit, lateral to the nasolacrimal duct orbital opening, the origin of the superior oblique muscle is located in the posterior orbit, at the orbital apex. Thus, in order to apply its force vector with the proper direction (i.e. from lateral to medial and from behind forward), the tendon of the superior oblique muscle must change direction. This is achieved by coursing around a cartilaginous pulley which is located at the superomedial angle of the anterior orbit. In doing so, the tendon makes a 51–55° angle (Freddo and Chaum 2018; Bertelli 2019). It is evident that the superior oblique muscle operates properly only if its angulated course is respected.

**Fig. 1:**
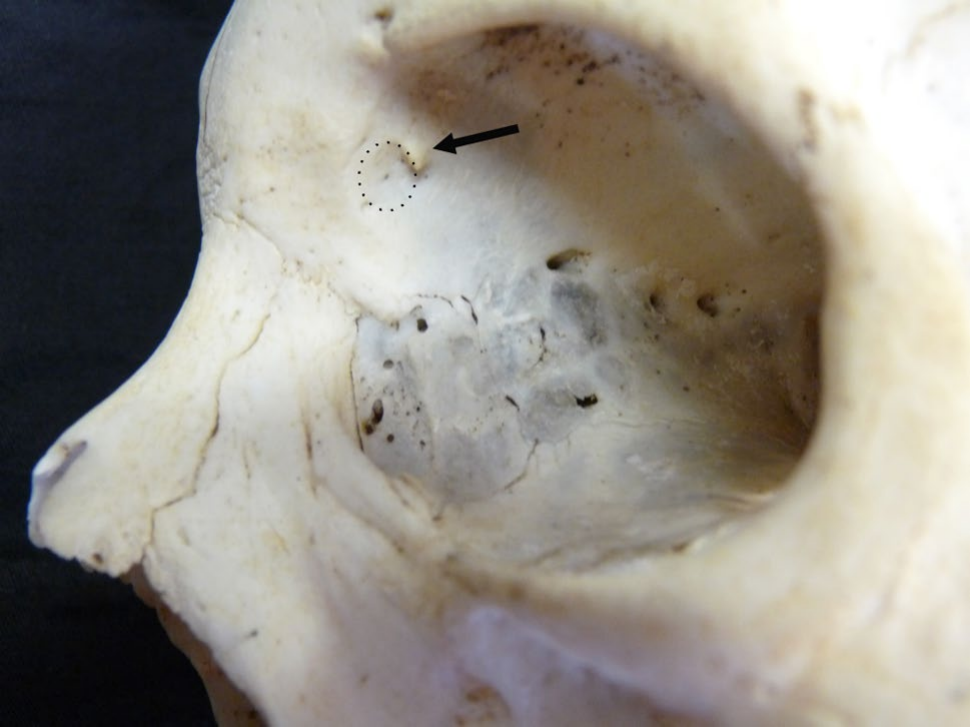
Left bony orbit. Examples of trochlear fovea (framed area) and trochlear spine (arrow). The trochlear spine arises from the posterosuperior edge of the trochlear fovea

The trochlea of the muscle is secured to the orbital wall by a couple of very short fibrous ligaments that insert to the border of a shallow depression known as trochlear fovea (TF) (Bron et al. 2001). The TF, located about 4 mm behind the orbital rim, not far from the frontolacrimal suture, is described as a feature of the orbital roof (Bron et al. 2001) even though, geometrically speaking, it belongs more to the medial than to the superior wall. Sometimes, a short and pointed bony process, the trochlear spine (TS) also known as spina trochlearis, arises from the edge of the TF (Fig. 1) acting as an insertion point for one of the ligaments that secures the trochlea to the orbital wall (Whitnall 1921). Frequency and topography of this bony process have been recently investigated in greater detail on dry skulls providing useful information for surgeons who need to operate in the superomedial region of the orbit (Aglianò et al. 2018). In this study, we aim to report observations carried out by computed tomography (CT) in order to complement Aglianò’s investigations with more reliable measures and to describe the relationships occurring between the TS and the tendon of the superior oblique muscle.

## Materials and methods

### Computed tomography

**Fig. 2:**
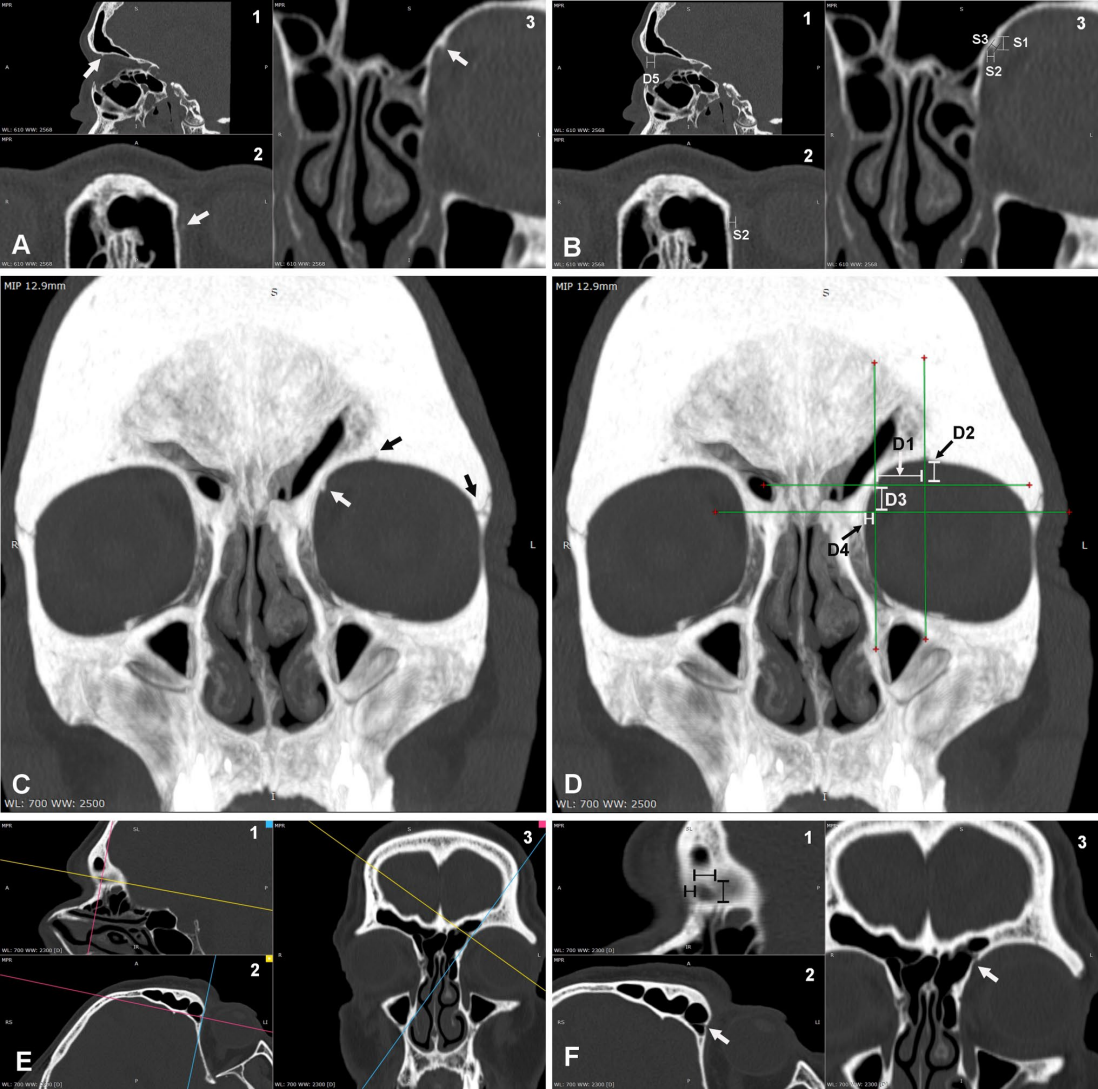
Morphometric methodology employed in this study for the trochlear spine and fovea. Procedures followed for their identification and measures. **A** Identification of the trochlear spine (arrows) in a left orbit by CT on the sagittal (A1), axial (A2) and frontal plane (A3). **B** Measurements taken on the planes shown in B: S1 = vertical projection of the spine tip from the orbital roof as seen in B3; S2 = axial projection of the spine tip from the medial wall as seen in B2 and B3; S3 = distance measured along a perpendicular line between the spine tip and the orbital wall as seen in B3; D5 = distance of the spine from the orbital rim as seen in B1. **C, D** Measurements taken according to Aglianò’s references (2018). In C it is shown the maximum intensity projection (MIP) tool that was applied to visualize a frontal slice of the skull as thick as 12.9 mm (in the present example) in order to observe on the same image both the trochlear spine (white arrow) and the bony landmarks, supraorbital notch/foramen and frontozygomatic suture (black arrows). **E, F** TF identification by CT on a left orbit. Once a small indentation adjacent to the superior oblique tendon reflection is observed on standard projections (a candidate for TF identification), the sagittal plane is reoriented to be tangent the surface of the orbital wall so that the TF can be seen as a small depression completely surrounded by a bony rim (E1). E2 and E3 show the sagittal plane tangent the TF whereas F shows the enlarged images of the planes seen in E. White arrows in F2 and F3 point to the trochlear fovea. In F3 the fovea is seen as a small pit surrounded by a bony rim. Diameters and distance from the orbital rim are taken as shown

A retrospective CT study in order to identify and characterize TS and small trochlear tubercles (TT)s located in the anterior orbit was carried out on sixty randomly selected patients (120 orbits from 33 females and 27 males with an average age of 58.9 ± 18.2 years) from the archives of the Unit of Interventional Neuroradiology (Siena). This study was approved by the Ethical Regional Committee for the clinical experimentation of the Tuscany region” and have been performed in accordance with the ethical standards laid down in the 1964 Declaration of Helsinki and all subsequent revisions. Patients were examined by CT for several reasons including trauma, nasal polyposis, sinusitis and tumours. Exclusion criteria comprised cases in which pathologic events involved the anterior orbit (e.g. tumours and serious traumatic injuries). CT scans were achieved with a GE Lighspeed VCT 64 slices (General Electric Healthcare Italia, Milano, Italy). Spiral scanning was performed at 140 kVp with “smart mA” function for thickness of 0.625 mm or 1.250 mm. Post-processing of the CT scans was carried out with RadiAnt DICOM viewer (version 2025.1) software. The presence of TS or TT was confirmed with the 3D volume rendering function of the software and measures were taken employing the multiplanar reconstruction function (MRP) (Fig. 2A). In particular, we measured the following parameters to define its size: TS vertical (S1) and horizontal (S2) projections into the orbital cavity along frontal and axial planes respectively; TS maximal height (S3) from the orbital wall along a line starting from the spine apex and perpendicular to the orbital wall (Fig. 2B).

In addition, we took measures of the distances between the TS/TT and superficial reference points projected on a frontal plane. In order to achieve that, we took advantage of the maximum intensity projection (MIP) tool of the software. A set of parameters were considered to devise a good system of coordinates for placing the TS/TT relative to superficial reference points projected on a frontal plane as previously reported (Aglianò et al. 2018). Briefly: D1 was the distance between the sagittal plane passing through the TS/TT and the sagittal plane intersecting the middle of the supraorbital notch/foramen; D2 was the distance between the supraorbital notch/foramen and the axial plane passing through the TS/TT; D3 was the distance between the axial plane intersecting the TS/TT and the axial plane passing through the frontozygomatic suture; D4 was the distance between the medial orbital wall and the sagittal plane passing through the TS/TT (Fig. 2C–D). In addition to D1-D4 parameters, we also measured D5, as the distance between the TS/TT and the orbital rim on a sagittal plane (Fig. 2B) and D6 as the distance between the TS/TT tip and the eye as measured along a frontal plane.

The identification of the TF was assessed by MRP. In particular, when a small depression was observed where the superior oblique tendon reflected, the sagittal plane was reoriented to be tangent to the orbital wall (Fig. 2E). When, under these conditions, a roundish depression could be observed along the reoriented sagittal plane, we considered it as a TF and we recorded vertical and horizontal diameters as well as the distance of its anterior edge from the orbital rim (Fig. 1F). For each parameter, we calculated mean ± standard deviation.

### Anatomic samples

In order to assess if the TS/TT is a feature that appears early, during the foetal stage of development, we analysed the collection of 360 foetal skulls housed at the Anatomical Museum of the University of Siena (Department of Medicine, Surgery and Neurosciences) whose details have been previously reported (Bonente et al. 2024).

### Statistical analysis

Statistical analysis with the Chi-square test was carried out to verify if there were significant differences between TS, TS + TT_1_ or TF frequencies in male and female skulls. Chi-square test was employed to determine if, for the same items, there was any side-related difference. On the other hand, two-tailed Student *t*-test was applied to compare linear measurements (e.g. size of the TS, diameters of the TF, distances of TS, TT_1_ or TF from the orbital rim) taken in two different groups (males vs. females, right vs. left).

In all cases, statistically significance was considered with *p* < 0.05.

## Results

### Trochlear spines and trochlear tubercles

CT allowed us to observe the inconstant presence of small bony processes, in the form of pointed TSs or of smoother TTs, at the superomedial angle of the anterior orbit.

**Fig. 3:**
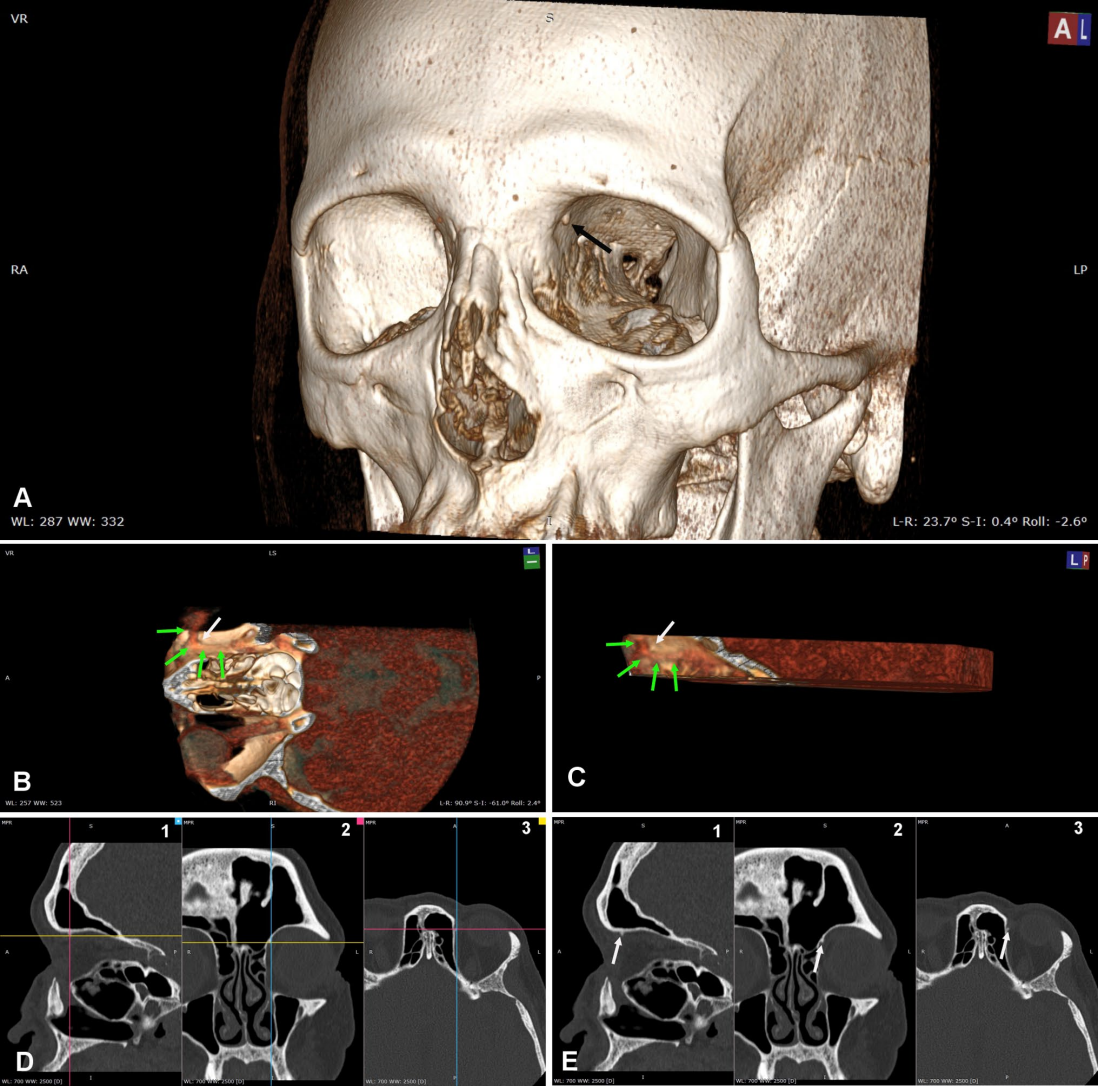
CT of a left orbit with a trochlear spine. **A** A prominent trochlear spine can be seen (arrow). **B, C** The adjustment of the image window allows to visualize intraorbital structures. To observe the superior oblique and its tendon (green arrows), lower and upper planes were clipped away as well as the lateral part of the orbit and the eye. B is an inferior view slightly tilted to the left. The superior oblique (green arrows) can be seen passing medial to the trochlear spine (white arrow) before bending to run towards the eye. In C the same object has been rotated to be seen from the left and slightly from behind. The superior oblique (green arrows) can be seen passing under the trochlear spine (white arrow). **D, E** The same trochlear spine can be seen in MRP. D demonstrate the orientation of the planes intersecting the spine. D1 shows the orientation of the frontal (magenta) and axial (yellow) planes as seen on the sagittal plane; D2 shows the orientation of the sagittal (blue) and axial (yellow) planes as seen on the frontal plane; D3 shows the orientation of the frontal (magenta) and sagittal (blue) planes on the axial plane. E is the same image seen in D without showing the planes to better visualize the spine (white arrows) on the sagittal (E1), the frontal (E2) and the axial (E3) planes

A true TS was observed in 12 orbits (10% of cases) (Fig. 3). As TS was found bilaterally in 4 subjects and in other 4 individuals it was detected only on one side (2 TSs on the right side and 2 TSs on the left side), it did not show any side-related prevalence. Also, sex-related difference in TS frequency was not significative with TS observed in 6 out of 33 females and in 2 out of 27 males. Data on the topography and on the size of the TS are detailed in Table 1 which shows that TS tip, on average, projected into the orbit for 2.0 mm on a sagittal plane (S1), 1.70 mm on an axial plane (S2) and 1.46 mm along a line perpendicular to the orbital wall (S3).

**Table 1:** Size and topography of bony processes on the superomedial angle of the orbit

	Orbits	S1	S2	S3	D5	D6
TS	12	2.03 ± 0.80	1.70 ± 0.45	1.46 ± 0.49	5.48 ± 0,71	6.61 ± 1.28
TT1	6	1.31 ± 0.54	1.17 ± 0.14	0.88 ± 0.39	5.15 ± 0.93	6.65 ± 1.56
TS + TT1	18	1.79 ± 0.79	1.525 ± 0.45	1.27 ± 0.52	5.37 ± 0.78	6.62 ± 1.33
TT2	2	2.25 ± 0.63	0.95 ± 0.29	1.3 ± 0.28	< 0.5	7.5 ± 0.99

S1 = vertical projection of the TS/TT into the orbit; S2 = horizontal projection of the TS/TT into the orbit; S3 = height of the TS/TT as measured along a line perpendicular to orbital wall; D5 = distance between the TS and the orbital rim; D6 = distance between the tip of the TS and the eyeball along the frontal plane

Additional data on TS topography were gathered by taking D1-D4 measures (see materials and methods). Results, with a comparison to previously published studies, are reported in Table 2.

**Table 2:** Topography of the TS compared with previously published articles

	D1	D2	D3	D4	D5
Aglianò et al. (2018)	8.5 ± 2.3	5.1 ± 1.3	5.7 ± 1.5	0.9 ± 0.4	4.2 ± 0,11
Atta et al. (2020)	7.22 ± 0.93	4.86 ± 0.93	6.14 ± 0.83	0.98 ± 0.48	3.77 ± 0.73
Present study	8.55 ± 2.91	4.39 ± 0.7	7.78 ± 1.97	3.39 ± 1.04	5.37 ± 0.78

D1 = distance between sagittal planes passing through the TS and the middle of the supraorbital notch/foramen; D2 = distance between the supraorbital notch/foramen and the axial plane passing through the TS; D3 = distance between the axial planes intersecting the TS and the frontozygomatic suture; D4 = distance between the medial orbital wall and the sagittal plane passing through the TS; D5 = distance between the TS and the orbital rim. Values are expressed in mm ± standard deviation

**Fig. 4:**
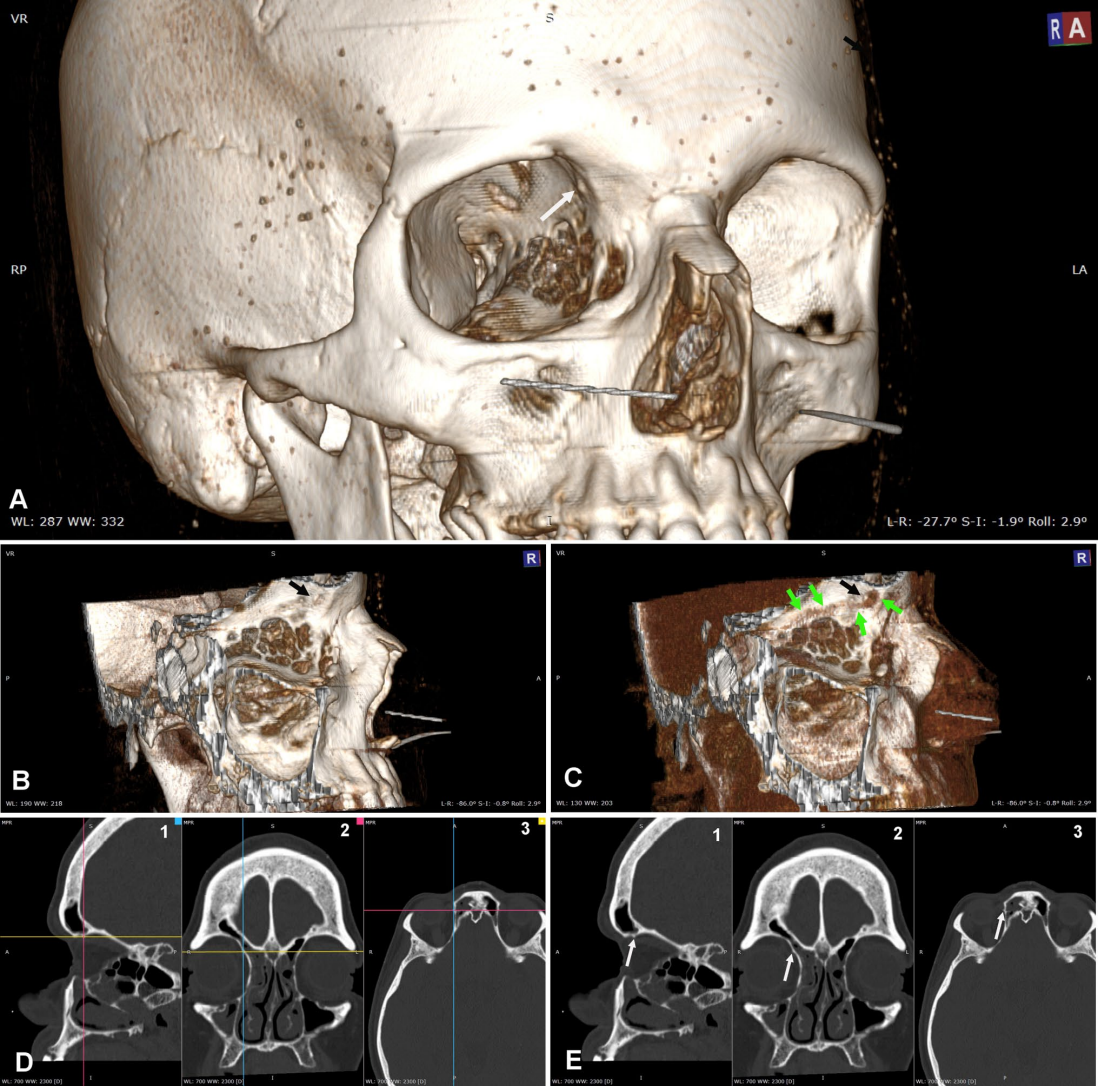
CT of a right orbit with a trochlear tubercle (TT_1_). **A** A TT_1_ can be seen within the orbit (arrow). **B, C** Lateral view of the orbit with the lateral wall and the orbit content clipped away for a better visualization of the medial wall and of the TT_1_ (black arrow). The adjustment of the image window in C allows to see the shade of the superior oblique muscle and tendon that courses under the TT_1_. **D, E** The same TT_1_ can be seen in MRP. D demonstrate the orientation of the planes intersecting TT_1_. D1 shows the orientation of the frontal (magenta) and axial (yellow) planes as seen on the sagittal plane; D2 shows the orientation of the sagittal (blue) and axial (yellow) planes as seen on the frontal plane; D3 shows the orientation of the frontal (magenta) and sagittal (blue) planes on the axial plane. E is the same image seen in D without showing the planes to better visualize TT_1_ (white arrows) on the sagittal (E1), the frontal (E2) and the axial (E3) planes

In six additional female orbits (5% of cases), the TS was replaced by a TT_1_ (Fig. 4). Statistical analysis, comparing prevalence of TS and TT_1_ in the two sexes, demonstrated a significative difference (*p* < 0.05) in favour of female individuals only by pooling together TSs and TT_1_s. In contrast, side-related difference did not come out either when considering TSs or TT_1_s alone or pooling them together. All measures related to the size (S1-S3) showed that TT_1_ was smaller than TS (Table 1). With good approximation, TT_1_ was located about the same spot as TS, 5.15 ± 0.93 mm from the orbital rim and 6.65 ± 1.56 mm from the eye. Indeed, with regards to the relationships with the tendon of the superior oblique, both TS and TT_1_ lied above and behind its sharp bending (Figs. 3, 4).

**Fig. 5:**
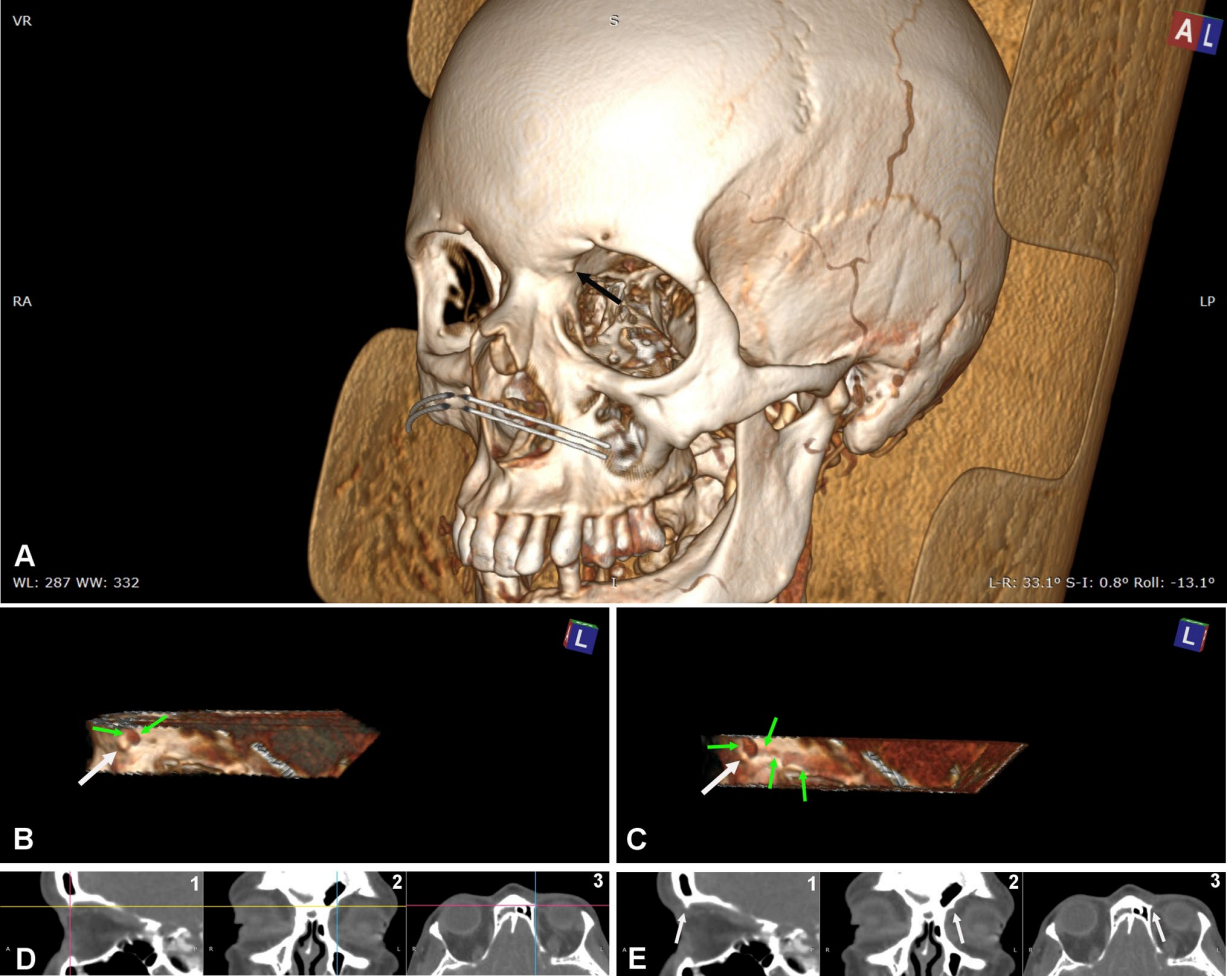
CT of a left orbit with a trochlear tubercle (TT_2_). The location of TT_2_ appears different from TT_1_. **A** A TT_2_ can be seen within the orbit (arrow). **B, C** Lateral view of the orbit with the lateral wall and the orbit content clipped away for a better visualization of the medial wall with associated tubercle (white arrow). The adjustment of the image window in C allows to see the transversely cut reflected part of the tendon (green arrows) located over and behind TT_2_. A different adjustment of the image window in D allows to see the shade of the superior oblique muscle and tendon that courses over and behind TT_2_. **D, E** The same TT_2_ can be seen in MRP. D demonstrate the orientation of the planes intersecting TT_2_. D1 shows the orientation of the frontal (magenta) and axial (yellow) planes as seen on the sagittal plane; D2 shows the orientation of the sagittal (blue) and axial (yellow) planes as seen on the frontal plane; D3 shows the orientation of the frontal (magenta) and sagittal (blue) planes on the axial plane. E is the same image seen in D without showing the planes to better visualize TT_2_ (white arrows) on the sagittal (E1), the frontal (E2) and the axial (E3) planes

Two further orbits (1.67% of cases) showed a TT characterized by a different topography (TT_2_) as it was situated just in front and below the site of the superior oblique tendon bending (Fig. 5). This quite rare bony process projected into the orbit for 0.95 ± 0.29 mm on an axial plane, 2.25 ± 0.63 mm on a frontal plane and 1.3 ± 0.28 mm along a line perpendicular to the orbital wall. The more advanced position of TT_2_, compared to TT_1_, was confirmed by MRP which demonstrated that its distance from the orbital rim was negligible (< 0.5 mm) and that it was located further from the eye (Table 1).

Analysis of 360 foetal skulls of various gestational age starting from the 17th week did not show any TS/TT.

### Trochlear fovea

In 31 orbits (25.83% of cases), the area involved by the presence of a TS or TT was characterized by a shallow depression referred to as TF. The TF could be visualized in 18 right orbits and 13 left orbits without any significative side-related difference. The 18 TFs on the right side were equally divided between males and females and the 13 left TFs were found in 6 female orbits and in 7 male orbits. Statistics did not show any sex-related difference in their right/side distribution.

Though with the caveat of a good degree of approximation, we measured the horizontal and vertical diameters of the TF that on average resulted 4.16 ± 1.08 and 3.84 ± 0.97 mm respectively (4.04 ± 1.08 × 3.69 ± 0.85 mm on the right side and 4.32 ± 1.11 × 4.05 ± 1,12 mm on the left side). The distance of the TF anterior border from the orbital rim was on average 3.42 ± 0.97 mm (3.36 ± 0.8 mm on the right side and 3.51 ± 1.2 mm on the left side). TF diameters in male orbits (4.11 ± 0.98 × 3.76 ± 1.11 mm) were virtually equal to those measured in female orbits (4.21 ± 1.21 × 3.93 ± 0.81 mm). The distance of the TF anterior border from the orbital rim was 3.77 ± 1.06 mm in male orbits and 3.05 ± 0.71 mm in female orbits. Neither size nor distance from the orbital rim showed any significative difference between the right and the left sides or between male and female populations.

## Discussion

Generally speaking, though largely incomplete because of the many openings transmitting nerves and vessels (Regoli et al. 2016; Regoli and Bertelli 2017; Durante et al. 2024), the orbit is considered as a cavity with smooth walls. The only, and seldom recalled, exceptions are the spina musculi recti lateralis and the TS. The former, located in the posterior orbit and for a long time acknowledged as a feature of the lateral margin of the superior orbital fissure (Bron et al. 2001), is indeed a small process arising from the orbital plate of the greater wing of the sphenoid in at least 41% of orbits where it gives insertion to the rectus lateralis muscle and to the annulus of Zinn (Bonente et al. 2024). The TS, on the other hand, lies in the anterior orbit. Information on its exact topography and frequency are scanty and, for the most part, must be dug out from very old scientific contributions (Ledouble 1903; Whitnall 1921). Ledouble (1903), in particular, observed that TSs can be replaced by TTs and that two types of TS/TTs can be found: a superior one, on the posterosuperior edge of the TF, and an inferior one, on the anteroinferior edge of the TF. These results are quite similar to ours as we too have detected both TSs and TTs. However, in our CT series we have observed that the TS is always located posterosuperiorly to the TF. In contrast, as TTs can be found in two different positions, we have referred to the posterior one as TT_1_ and to the anterior one as TT_2_. Actually, if we agree with Ledouble (1903), that implicitly considered TTs as mere variations of the TS, TT_1_ should be considered just as a different form of the superior TS and TT_2_ a variation of the inferior TS. The superior TS/TT_1_ and the inferior TS/ TT_2_ have been previously reported respectively in 15.95% and in 1–2% of orbits (Ledouble 1903). This frequency matches our own estimations (15% of orbits for the superior TS/TT_1_ and 1.67% for the inferior TS/TT_2_) and it is in good accordance with a more recent survey from Aglianò et al. (2018) (15.32% of orbits) who, however, did not make any distinction between superior and inferior TSs.

If the old works by Ledouble (1903) and Whitnall (1921) are a good source of information about the anatomic frequency, it is also true that they fall short as far as TS/TT size and topography are concerned. Indeed, they just report their position relative to the TF (i.e. posterosuperior o anteroinferior) and their greatest length (3 to 4 mm). Our surveying finds an average vertical length of 2.03 ± 0.8 mm with the longest TS that reached 3.78 mm. By pooling together TT_1_ and TSs, however, the average vertical length decreases to 1.79 ± 0.79 mm. As far as TS/TT_1_ topography is concerned, it is interesting to note the higher standard deviations of D1 and D3 compared to D2 and D4. This difference points towards a position of the TS/TT_1_ more stable, with respect to the orbit walls, than that of the landmarks (supraorbital notch/foramen and the frontozygomatic suture line) chosen as reference points. Indeed, it is known that the position of the supraorbital notch/foramen along the superior margin of the orbital rim has a remarkable degree of variation (Gupta 2008; Voljevica et al. 2022) as it is found 29.9 ± 3.6 mm (min 17.1 mm; max 37.8 mm) from the temporal crest of the frontal bone (Gupta 2008). Even the position of the frontozygomatic suture appears to vary considerably as it is located on average 8.17 ± 5.82 mm from Whitnall’s tubercle (Gayretli et al. 2023). In other words, when present, TS/TT_1_ should be considered a reference point more reliable than the frontozygomatic suture or the supraorbital notch/foramen.

A novel parameter, that we devised to outline TS/TT_1_ topography, is the distance of its tip from the eye as measured along the frontal plane (D6). D6 should not be considered as an indirect way to measure the length of the post-trochlear part of the superior oblique tendon. Indeed, the tendon is much longer having to embrace the eye to get to its insertion point on the posterior and superolateral quadrant of the eye. D6 is just a way to picture the room available to the surgeon who needs to approach the orbit medially and wants to respect the TS/TT_1_. As far as the TS/TT_1_ topography is concerned, we can compare our results (D1-D5) with previous investigations carried out on dry skulls using callipers (Aglianò et al. 2018; Atta et al. 2020). With the exception of D4, a remarkable good degree of concordance for D1-D3 and D5 can be seen by comparing the three studies (Table 2). The difficulty that investigators working on dry skull must have met to measure distances with a calliper between a bony reference point and virtual horizontal and vertical lines, however, is quite evident. Therefore, our measurements, taken with digital tools, are likely more precise than any previously reported result.

From a clinical viewpoint, the presence of the TS/TT may take on some relevance when it is required the surgical access to the medial wall of the orbit. In general, injury of the trochlea of the superior oblique muscle should be avoided in surgical procedures including frontal sinus trephination (Bartley et al. 2012), external fronto-ethmoidectomy (Graham 2016) and the superior lid crease approach to the anterior skull base (Gassner et al. 2016). To ensure proper superior oblique muscle movements after subperiosteal dissection of the orbital roof, it has been reported that the simple reapproximation of the soft tissues adjacent to the reconstructed orbital roof without reattaching the trochlea is all that is required (Haug 2000). However, superior oblique dysfunction resulting in transient or permanent diplopia has been observed on several occasions (Couch et al. 1990; Bartley et al. 2012; Grabe et al. 2013; Bischoff et al. 2020). Apart from more general considerations, it appears evident that, if periosteal reattachment of the trochlea on a flat surface is certainly a straightforward procedure, it may result trickier on a spiny surface.

A distinct TF, in the form of a small circular pitting, is visible in 80% of orbits (Whitnall 1921). This estimation is in good accordance with more recent works carried out on dry skulls that found the TF in 73.4% or 84.2% of orbits (Aglianò et al. 2018; Atta et al. 2020). Because of its shallow nature, however, it is easily understandable why, by CT, we could demonstrate it only in 25.83% of cases, a value that certainly underestimates its real incidence. Nevertheless, even though aware of a certain degree of approximation, we have measured TF diameters (4.16 ± 1.08 and 3.84 ± 0.97 mm) for which we did not find any previous record. In contrast, the distance of 3.42 ± 0.97 mm, measured between the anterior border of the TF and the orbital rim, is in good accordance with Whitnall’s report (1921) (from 3 to 4 mm).

According to Ledouble (1903), the superior and inferior TS/TT correspond to the insertion points of the fibrous ligaments that secure the cartilaginous trochlea to the orbital wall and that undergo a process of late calcification. The much higher frequency of the superior TS would be the result of the greater stress exerted by the superior oblique tendon on the corresponding ligament (Whitnall 1921). On the other hand, a chondral ossification center specific for the TS and the presence of a suture at its base have been also reported (Keibell and Mall 1910; Lang 1983), suggesting an early and genetically determined development of the TS. In this context, since the TS may attach to the frontal bone later, with the formation of a suture, the absence of the TS in foetal skulls cannot be considered diriment as developing TS could have been just gone lost. Determining the genetic involvement in TS/TT expression is not an easy matter. TS/TT is included among the nonmetric traits, also known as epigenetic variants, which are likely the result of a combination of polygenic determination and local environmental factors (Hauser and De Stefano 1989; Carson 2006). Taking advantage of a collection of 496 skulls distributed along four successive generations with known familial data, a statistical maximum-likelihood variance components analysis of heritabilities of cranial nonmetric traits, supports a considerable role of genetic in TS expression (Carson 2006). Interestingly, however, the same analysis did not find any sex-related TS expression difference. This is in contrast with our own findings which point towards a significative prevalence of TS/TT_1_ in female orbits.

In conclusion, the present study supplies the first digital evaluation by CT of the TS/TT topography in the human orbit. It also establishes the existence of two types of TS/TT with different relationships with the superior oblique tendon that likely represent the insertion points of the trochlear ligaments.
